# 185. Nirsevimab Binding Site Conservation in RSV F Protein between 2019 and 2024: Analysis of Sequencing Data from the US OUTSMART-RSV Surveillance Study and Public Databases

**DOI:** 10.1093/ofid/ofaf695.063

**Published:** 2026-01-11

**Authors:** Kelly Ann Mahool, Gustavo H Kijak, Bahar Ahani, Tyler M Brady, Amy Nguyen, Emma Schaefer, Sarah R Sincero, Katie Streicher, Kevin M Tuffy, Deidre Wilkins

**Affiliations:** AstraZeneca, Gaithersburg, Maryland; AstraZeneca, Gaithersburg, Maryland; AstraZeneca, Gaithersburg, Maryland; AstraZeneca, Gaithersburg, Maryland; AstraZeneca, Gaithersburg, Maryland; AstraZeneca, Gaithersburg, Maryland; AstraZeneca, Gaithersburg, Maryland; AstraZeneca, Gaithersburg, Maryland; AstraZeneca, Gaithersburg, Maryland; Translational Medicine, Vaccines & Immune Therapies, BioPharmaceuticals R&D, AstraZeneca, Gaithersburg, MD

## Abstract

**Background:**

Nirsevimab is an extended half-life anti-respiratory syncytial virus (RSV) fusion (F) protein monoclonal antibody licensed for the prevention of RSV lower respiratory tract disease in neonates, infants, and medically vulnerable children. Vigilant surveillance programs are required to monitor the conservation of the nirsevimab epitope between RSV seasons due to potential viral evolution. OUTSMART-RSV is an ongoing multi-center, prospective, molecular surveillance study to monitor the prevalence and distribution of RSV strains in the US and track the emergence of RSV F variants and their susceptibility to nirsevimab neutralization.

**Figure:**
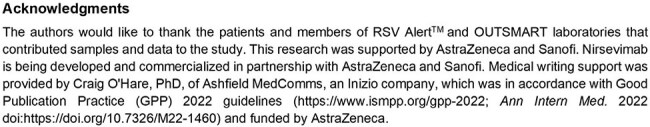

**Figure:**
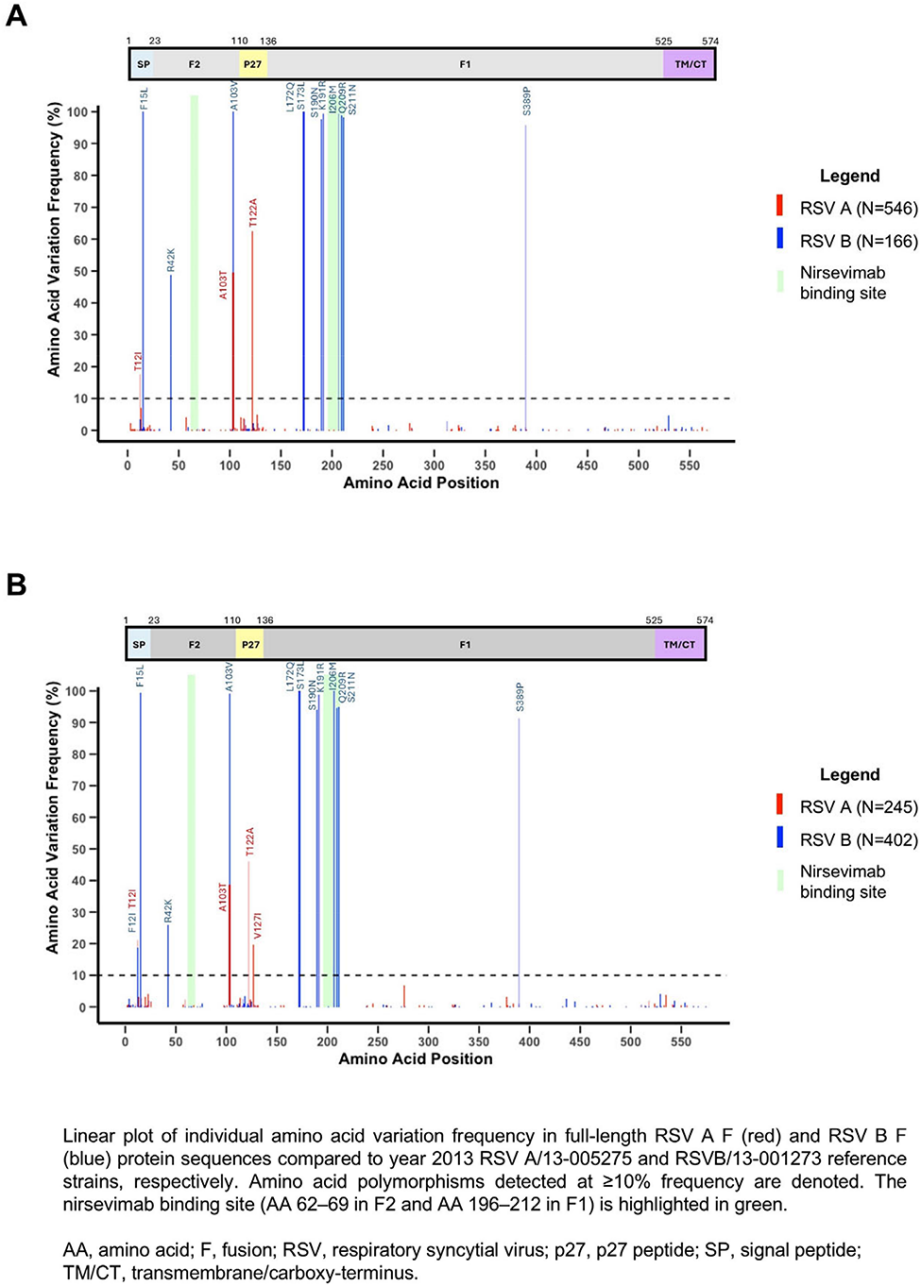

**Methods:**

Nasal swabs were collected from infants and adults seeking medical attention for a respiratory infection. RSV-positive isolates were analyzed using next-generation sequencing and subtyped based on the hypervariable region of the G protein. F protein polymorphisms were identified by comparison to RSV A and B reference sequences. Concordance was assessed between F protein sequences from OUTSMART-RSV and sequences uploaded to NCBI and GISAID databases from 47 countries between 2019 and 2024. F protein variants with increased prevalence were phenotypically evaluated via reverse genetics rescue and *in vitro* microneutralization assays.

**Figure:**
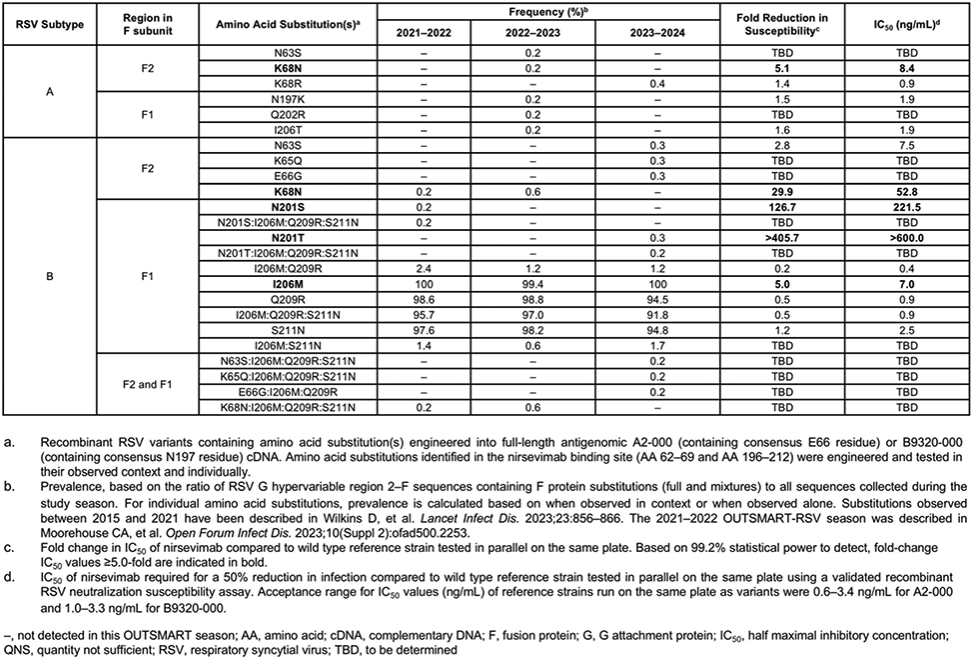

**Results:**

A total of 1,359 RSV F sequences were available for analysis (2022–23, n=712; 2023–24, n=647). Genetic diversity of RSV F sequences was low compared to reference sequences. Amino acids in the nirsevimab binding site were > 99% conserved in 25/25 positions in RSV A and 22/25 positions in RSV B (Figure 1). High concordance was observed between OUTSMART-RSV F protein sequences and those from NCBI and GISAID (N=10,167).

Nirsevimab retained neutralization activity against all recombinant RSV A F variants containing nirsevimab binding site substitutions in their observed context (Table 1). Known nirsevimab resistance substitutions in RSV B were rare (i.e. < 1% of sequences) with no increase in prevalence by the end of the analysis (i.e. the 2023–24 season).

**Conclusion:**

The nirsevimab binding site remains well conserved among global RSV A and B strains and the prevalence of variants containing resistance-associated substitutions was very low. Nirsevimab continues to neutralize > 99% of RSVs in circulation.

**Disclosures:**

All Authors: No reported disclosures

